# Periods of uncertainty: The experience of at‐risk young adult Arabs during the transition to adulthood in the wake of COVID‐19

**DOI:** 10.1111/cfs.12927

**Published:** 2022-04-23

**Authors:** Yafit Sulimani‐Aidan

**Affiliations:** ^1^ Bob Shapell School of Social Work Tel Aviv University Tel Aviv Israel

**Keywords:** COVID‐19, national minority, transition to adulthood, young adult Arabs

## Abstract

The ongoing COVID‐19 global health crisis has both short‐ and long‐term implications for the lives of young adults worldwide, especially young adults from vulnerable communities. The current exploratory study is the first, to our knowledge, that investigates the impact of the pandemic on the lives of at‐risk young adult Arabs, who are part of a national minority group in Israel. Twenty‐eight at‐risk young adults aged 18 to 25 participated in semistructured interviews regarding the experiences, challenges and barriers they faced as emerging adults during the pandemic. Grounded theory analysis and theoretical thematic analysis were used to analyse the interviews. Findings revealed that the pandemic and the policy decisions made in its wake influenced the young adults' lives in primary areas including their financial and occupational status, their social ties and social support networks, their relationships with their biological families and their future plans and goals. The study's findings shed light on the complex reality of at‐risk young adults during the pandemic and emphasize the increasing vulnerability of this cohort. The challenges they face as members of a collectivist society and of a national minority group are discussed. Implications for policy and practice highlight the need to increase these young adults' occupational and financial opportunities and to design holistic services that take into account their personal and sociocultural characteristics.

## 1 INTRODUCTION

### 1.1 COVID‐19: Implications for emerging adults

COVID‐19, the disease caused by the novel coronavirus SARS‐CoV‐2, was characterized as a pandemic in March 2020. This pandemic has affected all aspects of human life worldwide and has manifested in increasing numbers of infected people and a rising mortality rate in many countries (WHO, 2020). The COVID‐19 pandemic has caused an unprecedented crisis on almost every level and has affected lives in many ways. To prevent the spread of the disease, many governments have imposed measures such as social distancing and home confinement (Wang et al., 2020).The Israeli policy regarding the pandemic has been quite strict from the start. Comprehensive restrictions such as avoiding public spaces, staying home except when absolutely necessary to go out (i.e. work, in accordance with prescribed limits; buying food or medicine; receiving medical treatment) and not congregating have all been imposed. All educational institutions for children ages 0–18 were shuttered (until mid‐May 2020) including higher education facilities. Restaurants and entertainment venues, shops of all kinds, malls and shopping centres, hotels and many others businesses (excluding those defined as essential, such as grocery stores and pharmacies) were forced to close immediately. Non‐essential industries were permitted to employ only a limited number of workers. Furthermore, starting in April 2020, the Israeli government announced three periods of curfew and a 100‐m limit on travel from home for nonessential activities (Ministry of Health, 2020).

These decisions have been considered effective in preventing the spread of the disease and decreasing the rate of infection, hospitalization, and morbidity. However, home confinement and curfew are a great cause of concern as they have impacted people's lives globally in different areas including employment and economic status, social life, physical health and mental health (Pieh et al., 2020). Recent studies on the impact of COVID‐19 have shown that the pandemic has led to increased unemployment levels, social isolation and an increase in suicide rates (McIntyre & Lee, 2020; Pieh et al., 2020; Wang et al., 2020). Accordingly, the mental well‐being and quality of life of many people worldwide has been influenced dramatically by the pandemic's implications (Kilani et al., 2020).

Although more research is needed in order to fully understand the implications of the pandemic for the lives of at‐risk young adults in particular, studies examining the impact of the pandemic on young adults in general suggest that this population is extremely vulnerable to its implications. For example, Ranta et al. (2020) found that young people were more concerned about the effects of the COVID‐19 pandemic on their mental well‐being, career and studies and economic situation than were older people. Also, a recent survey conducted during the pandemic's peak highlighted significantly higher stress levels and elevated anxiety and depression symptoms among young adults in comparison with older respondents (Czeisler et al., 2020). Furthermore, young adults who became unemployed due to the coronavirus outbreak reported increased psychological distress, higher financial strain and loneliness (Achdut & Refaeli, 2020).

These short‐ and long‐term implications could have a particularly strong effect on the lives of at‐risk young adult Arabs who belong to a national minority group in Israel facing social and economic marginalization, lower resources and social discrimination and exclusion (Chaider et al., 2010; Miaari & Hadad Haj‐Yahya, 2017). However, the consequences of COVID‐19 and the resultant policy decisions for the life course of these vulnerable young adults as they navigate toward adult life are still unknown.

### 1.2 At‐risk young adult Arabs in Israel during the transition to adulthood

At‐risk young adult Arabs in Israel suffer from persistent shortages or distress in one or more of the following areas: education, employment and skills; well‐being and emotional health; food security, health and protection; and social and familial affiliation (Ministry of Labor, Social Affairs and Social Services, 2020). As part of a national minority, they are exposed to social exclusion, discrimination and oppression (Naber, 2005; Shoshana, 2016). A high rate (43%) of young adult Arabs (18–24) live with their families in poverty. Also, 40% of the Arab young adults in this age group are unemployed, and 20% are not in frameworks of employment, education or training (Central Bureau of Statistics, 2019). In addition, young adult Arab women are vulnerable to experiencing abuse or violence at the hands of their family members, as well as aggression from their community (Shalhoub‐Kevorkian & Daher‐Nashif, 2013).

This reality places them under a myriad of stressors and risks during the transition to adulthood, a period during which significant and future‐shaping decisions in central spheres of life are made (Arnett, 2004). The vulnerability of this cohort has intensified in the face of the global COVID‐19 pandemic that has generated multiple challenges for societies, increasing economic‐psychosocial problems for social service users (Walter‐McCabe, 2020).

Emerging adulthood is a critical period of transition, during which young people are tasked with establishing adult identities and striving for stability in domains such as housing, employment, career, marriage and new social networks (Arnett, 2004; Berzin et al., 2014). According to the emerging adulthood theory, this period represents an important developmental phase that requires coping with complex instrumental and developmental tasks, during which young adults optimally acquire the skills and experiences that allow them to take on adult roles and responsibilities. This life stage is characterized by identity exploration, instability, self‐focus, a feeling of being ‘in between’ as well as the perception of a range of possibilities (Arnett, 2000). The theory of emerging adulthood postulates that although this period is frequently fraught with challenges, it is also a time of great hope and potential, in which young people perceive and pursue opportunities to build higher levels of stability in their relationships, routines and resources (Arnett, 2004).

Like their peers in the general population, at‐risk young adult Arabs must also contend with transition‐to‐adulthood issues, including career, school and family. However, the few studies examining the transition‐to‐adulthood experience of at‐risk young adult Arabs, pre‐COVID‐19, found that they as part of a minority group experience additional challenges. On top of the turbulence of emerging adulthood, they, as part of a collective society, have certain obligations to family and community (Ibrahim & Howe, 2011; Sulimani‐Aidan, 2020a) and must cope with low socioeconomic status and poor personal and environmental assets (Ibrahim & Howe, 2011; Phinney, 2006). In addition, Israeli‐Arab young adults may be particularly preoccupied with their self‐identity as a minority group, the political circumstances surrounding them and feelings of discrimination (Miaari & Hadad Haj‐Yahya, 2017). These aspects may prevent them from exploring options for themselves or pursuing their goals (Sulimani‐Aidan, 2020a).

Against this backdrop, it is important to understand these young people's transition to adulthood in light of the ongoing global pandemic. A study conducted among the Arab minority in Israel during the first COVID‐19 wave found that this population experienced higher stress levels than did other non‐minority populations, potentially harming their mental health (Satran et al., 2021). To date, however, no study to our knowledge has investigated the subjective experience of at‐risk young adult Arabs in Israel during this pandemic. In response, the present study investigated the challenges and barriers in the lives of these vulnerable young adults at a time of great social uncertainty, namely, the global COVID‐19 pandemic.

## 2 RESEARCH GOALS

In this exploratory study, we aimed to investigate the experiences, challenges, and barriers faced by at‐risk young adult Arabs during the transition to adulthood and under the circumstances posed by the COVID‐19 pandemic. In the current study, this critical time of uncertainty and concurrent individual transition was conceptualized within the framework of emerging adulthood theory (Arnett, 2000, 2004).

The study's findings could shed light on the unique characteristics and needs of at‐risk young adults from various ethnic groups and fill the gap in the literature regarding these young people's paths to adulthood, especially in light of the continuing global health crisis. The findings might also help policy makers and service providers gain a better understanding of the way to support these young people during this crucial transitional period to adult life and strengthen their resilience and outcomes as adults despite the pandemic's negative effects.

## 3 METHODS

### 3.1 Participants

The most significant ethnic division in Israel is between the Jewish majority and the Arab minority, with the latter comprising about 21% of the population in Israel. About 85% of the Arab population are Muslim‐Arabs, 7.4% are Druze and 7.2% are Christian‐Arabs (Central Bureau of Statistics, 2019). The study's sample included 28 Israeli‐Arab young adults, mostly Muslims, between the ages of 18–25 (*M* = 22) who utilized one of the social welfare services throughout the country. Of the participants, 14 (50%) were young adult men and 14 (50%) were young adult women. The majority were single (92%), and two were divorced. Also, prior to the pandemic outbreak, most of the participants worked in non‐professional and/or temporary positions including in restaurants (e.g. as waiters), supermarkets or manufactories. However, at the time of the interviews, most of the participants (75%) were unemployed or furloughed due to the lockdowns. Four of the participants were integrated in different educational frameworks, such as specialized colleges where they were receiving training for a specific vocation. In terms of housing, the majority of the participants lived in their parents' homes or with members of their extended families (e.g. an older sibling or the grandparents). Five of the participants who had previously been living in rented apartments returned to live with their parents. Their risk backgrounds were varied and included economic hardship, educational gaps, histories of abuse and neglect and social isolation. Also, six of the participants had a history of living in residential care.

### 3.2 Procedure

After obtaining approval from the ethics committee of the researchers' universities, the research staff received contact information from caseworkers who work with at‐risk Arab young adults in social welfare services throughout the country. Only young adults who agreed to take part in the study by their social workers were included. The researchers then randomly selected a convenience sample of young adults between the ages of 18 and 25 who were approached over the phone, given an explanation of the study's goals, and asked for their written consent to participate in the study. Of the 35 young adults, 28 matched the selection criteria that were defined ahead of the interview and gave their consent to participate. The selection criteria were as follows: young adult Arabs in the early years of their emerging adulthood (i.e. ages 18–25), from a variety of frameworks—that is, in professional training, employed or unemployed—living in both rural and urban areas.

A semistructured interview protocol was developed, consisting of open‐ended questions. All interviews were conducted in Arabic, recorded and transcribed and then translated into Hebrew. The participants were interviewed individually via Zoom due to COVID‐19 restrictions. Each interview lasted between 35 and 60 min, during which time the participants were asked to describe themselves (background, occupation, age, etc.). They were also asked about their current life status and the challenges and barriers they faced in their daily lives in light of the pandemic.

### 3.3 Data analysis

The methods used to analyse the interviews were grounded theory analysis (Charmaz, 2006; Corbin & Strauss, 2014) and theoretical thematic analysis (Braun & Clarke, 2006), which are often used to identify patterns and themes within qualitative data. The aim of grounded theory is to help us understand phenomena holistically, by examining individual perceptions and meanings and their relations with broader interpersonal and social processes and environmental contexts (Corbin & Strauss, 2014). In addition, theoretical thematic analysis allows for the use of pre‐existing theoretical frameworks, such as Arnett's emerging adulthood theory (Arnett, 2000).

Analysis was performed by three readers (the two interviewers and the leading researcher) who interpreted the themes, which expressed participants' perceived challenges and resources. The readers employed an incident‐by‐incident coding technique in which every portion of the interview transcript was read and coded for important themes. This phase was followed by a focused coding process, during which the incident codes were reread and analysed in order to identify larger themes. During these phases, the readers analysed the interviews and then met to discuss the themes and resolve any discrepancies. Finally, after the challenges and resources codes had evolved, the readers once again sifted through all the data, using a focused coding process (Corbin & Strauss, 2014). The ensuing coding created three main categories with various related subcategories. To further check the validity of the findings, a summary report was translated into Arabic and provided to the participants via email, and their feedback was integrated into the final data analysis.

## 4 FINDINGS

### 4.1 Experience during COVID‐19

Four major themes arose from the participants' descriptions. The first theme was their *financial and occupational barriers* which included their struggles with past and present economic hardships and occupational challenges in light of the pandemic. The second theme concerned the effect the pandemic had on their *relationships with their biological families*, which in many cases were complex and, in some cases, had already been abusive even prior to the pandemic. The third theme included the impact of the pandemic on their *social ties* and concerned both a sense of loneliness and social isolation, as well as a lack of support and guidance from adult figures, which they very much needed specifically during these challenging times. Finally, the fourth theme concerned the impact of COVID‐19 on the participants' *future plans and goals*. This theme relates to the many sudden changes in plan that were forced on them or their inability to pursue their educational or professional goals as a result of the pandemic. It is worth mentioning that most of the participants did not mention their mental health or emotional distress as a theme in and of itself. However, they described in detail the negative effect that all four of the themes had on their emotional situation and linked their feelings with the changes in their financial status, social networks and future perceptions.

### 4.2 Financial and occupational status

Participants' financial status and occupational opportunities emerged as one of the main areas affected/damaged by the pandemic. The majority of the participants lost their jobs:

“The pandemic had a major influence on my life. Lockdowns and face masks and the isolation all had a negative effect on me. But I think that the fact that I was forced to leave my work was the worst thing for me. I spent most of my time there and I loved working there” (woman, 23).

Other participants described their difficulties in finding work in light of the virus's spread and governmental restrictions, as this young man (24) said:

“The most challenging thing for me now is that I do not have a stable job. The lockdowns and the closings of many things like restaurants, hotels, and stores. This makes it hard to find a permanent job.”

Those who wished to apply for the few jobs that were available found that their relatively poor mastery of the Hebrew language posed a strong barrier:

“This period is very challenging and difficult. I find it very hard because I was fired and cannot find a job. All the jobs require good Hebrew and I do not have it. Especially during COVID‐19, there aren't so many employment opportunities” (man, 19).

The loss of their jobs impacted the financial status of these vulnerable young adults, which in many cases was weak to begin with. Many of them struggled daily to provide for themselves. As opposed to other young adults (i.e. from the non‐Arab, non‐at‐risk sector) who could lean on their families for financial support at this difficult time, the change of occupational status among these at‐risk Arab young adults had a crucial influence on their financial status. A young man (22) who was not in touch with his parents and lived with his grandmother lost his job at a clothing store and said:

“This period is very challenging. I have to work and earn money in order to survive. The store I worked at for two years closed. It is very hard for me financially. I cannot be a burden on my grandmother, who I live with.”

Two participants (women, 25 and 28) who were divorced and the only providers for their children said:

“Financial hardship is the most difficult thing. Due to COVID‐19 and the lockdown, I stayed at home for a long time without a job. But still, I have to take care of my children's daily needs and pay the bills. This period made me worry about my girls' future. What will their fate be?”

“Before the COVID‐19 pandemic I worked in a stable job but then I was fired and could not find another job. My financial status deteriorated and so I had to leave my apartment because I could not pay the rent. I now live with my sister. She accepts me and my children but I do not want to make it hard on her, and also, we need our own space.”

### 4.3 Relationships with family members

The majority of participants described the influence that the pandemic was having on their relationships with family members. They expressed concern regarding their family members' health, especially that of their parents and grandparents. Those who occasionally managed to find employment feared that they might infect their elders with the disease. However, at the same time, the lack of employment and the national lockdown imposed upon them a situation in which they were forced to interact more extensively with their families, and narrowed their freedom and privacy:

“I feel like I do not have my own space at home. I used to go out with my friends to drink coffee. Now I do not have this option and I do not have my own space. We are a big family in a very small house. That's why there are many tensions and quarrels. I have good relationships with my siblings, but the situation is so intense that we spend too much time together, so it is tense” (man, 22).

The intensity of the interactions, the large number of family members living together under one roof and the differences in needs and ages led to many conflicts. Also, participants who were forced to return to their parents' homes because they could not afford to pay their rent felt that their freedom and independence had been taken from them or that they were obligated to support their families. As one participant (man, 24) said:

“… Another thing that makes it very hard for me is my parents' roles. They interfere with everything I do. Who I go out with, when I will return home at night … I am not a child anymore, but they will not let me be fully independent as I used to be … either way, they are my parents… I must listen to what they say.”

Some participants had a history of abusive relationships with their families and, as a result of the pandemic, had no choice but to return home or contact their families more frequently during lockdowns. This situation increased their risk of negative interactions and hurt/maltreatment at the hands of their families. In most cases, it was the female participants who experienced more abuse and restrictiveness during this period:

“My father is a very angry man. He has always decided what's best for me. He controls everything in my life … we live together and I do not have siblings to be with. Especially now during COVID‐19 the country is shut down so we fight a lot, so many times, over so many things. But the hardest thing is that he behaves more violently toward me both physically and verbally” (woman, 23).

Another participant (woman, 21) who had been removed to residential care as a child, due to abuse by her parents, described her relationship with them today:

“I cannot leave home and escape the reality I am in right now. My mother has a history of using drugs. She is nervous because of the news and now she drinks more extensively. Her situation is deteriorating. My father was always violent toward us. He cannot deal with his feelings so he takes it out on us now … “

Another young woman (25) had begun divorce proceedings against her husband because of his abusive behaviour toward her, but due to lockdowns and restrictions, she was forced to live with him, and the pandemic aggravated their situation even further:

“I do not want to live with him but I do not have a choice … I have two daughters. He is very angry and humiliates me. It's very hard to live with him. In order to protect myself I sleep at an elderly woman's house in exchange for my helping her. It's worse now since the coronavirus. He is always at home and I do not have a minute for myself.”

### 4.4 Social ties and loneliness

Most participants described a loss of social ties and sense of loneliness. They emphasized the way that their already weak social ties became even weaker (or were lost altogether) and their strong need for social support, especially during these challenging times. Participants who had experienced some sort of formal or informal support prior to the pandemic highlighted the importance of this support in their lives and the difficulty in coping without the support of these meaningful figures:

“My mother was always supportive of me. I grew up in residential care so I missed her a lot and now I want stay close to her. But I found a job in another city and I rent an apartment there. The coronavirus period made it very hard for me to cope since I could not meet with her. I am very attached to her, but I was afraid for her health, so we only spoke on phone … I also had to work … I did not want to jeopardize her health (man, 25).

Another participant who prior to the pandemic had visited the youth centre in his village on a regular basis, said:

“Being at home is very boring and hard. The atmosphere is unstable and I do not want to stay there. I used to be a D.J. but I cannot do it anymore so it affects my mood. Also, the youth center I used to go to is closed. They used to do all kinds of activities that were good for me, and the staff supported me…this place saved me from the street … but now I cannot go there anymore or meet my friends there or talk with my social worker.”

Many of the participants, as stated, had a weak social network even before the pandemic. However, for many of them, the consequences of the pandemic affected the few supportive relationships that they did have in different ways. The lack of social ties and sense of loneliness was described by this participant (woman, 26):

“If I ever thought that I had friends … today I certainly know that I do not have any. The coronavirus helped me understand it. I came to realize who's with me and who is against me. My dog is better than all of those people.”

Another participant (man, 24) said that the pandemic had prevented him from seeing the one person he wished to see, who lived in another city:

“My parents divorced when I was a child, and I am not in touch with my father … I never had friends in school … when I grew up, I realized I was attracted to men, but I was ashamed and afraid that people in the community and my family would find out. I have only one friend I enjoy being with … I feel that he believes in me and loves me…but one of the most difficult things in this period is that there are a lot of lockdowns and restrictions so I cannot see him…”

The situation of one participant (man, 26) who lived in a hostel (financed by his employer) reveals the way in which the pandemic could affect one's social life both directly and indirectly:

“I do not really have anything of my own. I feel that I am standing in one place. I started taking a class but it was stopped because of COVID‐19 so I do not have anything to do and I am alone most of the time. Sometimes I visit my friend but then I come back to an empty apartment. I had a roommate but she left and went back to live with her parents.” For other participants who had previously struggled socially, this period posed additional challenges that increased their sense of social isolation: “In these times I feel very lonely, without anyone who supports me, like family and friends. It was always hard for me to find new friends, but now during COVID‐19 it is much harder … I rarely meet people” (man, 20).

### 4.5 Future goals and plans

Participants with expectations and goals regarding their futures, as well as those already in the process of meeting those expectations and goals, described a negative change in relation to their future plans. The uncertainty and ‘survival mode’ attitude created in the wake of COVID‐19 affected their motivation to pursue goals, forced them to change their plans or held them back from reaching the goals they had set for themselves before the pandemic arrived. For example, some participants had planned to save money in order to begin their studies or rent an apartment but, because of the pandemic, were forced to continue living with or return to their families. As one participant (man, 22), who lost his job and had to move back in with his parents, said:

“Now with all the lockdowns and changing guidelines by the government, I feel very confused. I am very worried about my future. My parents cannot support me economically. I planned to save money so that I could have a profession that would help me find a better job in the future. But now I am not sure I will be able to do it because of COVID‐19.”

In some cases, participants had to postpone their plans because they did not have the means to realize them during the pandemic:

“The coronavirus affected many areas in my life, such as my work and studies. Although I enrolled in school, I wasn't sure how it could be done. I was afraid that classes might stop in the middle or that we would need to learn virtually via Zoom. But I do not have a computer at home, and I cannot afford one in my current situation. So I had to put off my studies” (woman, 22).

Some participants, who had already been financially supporting their families pre‐pandemic, now felt that the pressure to provide for their families had grown, further narrowing their own future options. One participant (woman, 22) worked as a cashier, lived with her seven siblings in poverty, and had a mother who struggled with mental illness. As she said:

“I have a very big responsibility to my family. I have always had this responsibility. That's why I was so afraid that I might lose my job. Because of COVID‐19 I had to stop my driving lessons. That was really frustrating. I felt that this was my way to advance and not stay in the same place all the time. I have so many dreams … its very challenging now.”

The pandemic's impact on the participants' financial status forced some of them to change their plans and concentrate on their families instead, a situation described by one young woman (22) in the following way:

“My goal was to save money in order to study. I saved money from an earlier job and planned to study to become a chef. My family was going through a very hard time financially because of the pandemic. That's why I had to change my priorities and put them at the top of my list. I put aside my plans and gave them the money I saved, so they could get through this challenging time. I cannot find a stable job at the moment so I work here and there and will try again to save money…”

## 5 DISCUSSION

The goal of the present study was to investigate the challenges and barriers experienced by at‐risk young adult Arabs during the COVID‐19 pandemic. It was informed by the emerging adulthood theory: a theory that views emerging adulthood as a critical period of transition, during which young people are tasked with establishing adult identities and striving for stability in domains such as housing, employment, career, marriage and new social networks (Arnett, 2004; Berzin et al., 2014; Williams & Sheehan, 2015). In line with this theory, we wished to gain an understanding of the life experiences of at‐risk young adult Arabs that might limit or challenge this primary transition‐to‐adulthood task during a global crisis.

### 5.1 Periods of uncertainty: The instability of emerging adulthood meets the uncertainty surrounding COVID‐19

The study's main finding revealed that the pandemic exerted an extensive influence on the lives of these young adults in many respects. Although none of the participants contracted the virus or suffered from the health implications themselves, the social and financial implications alongside governmental policy decisions had far‐reaching implications for their occupational and economic status, social support networks, family relations, and future plans. Specifically, the picture painted by this study suggests that at‐risk young adult Arabs' vulnerability has increased because of the pandemic. Indeed, the financial strain, unemployment and difficulties in finding new jobs led to their frustration and discouragement not only because of the impact of the pandemic itself but also because of their status in society and the limited resources and opportunities which became even more limited once COVID‐19 broke out. Similar challenges were document among other vulnerable group of young people. For example, Roberts, Mannay et al. (2021) found that during COVID‐19, care leavers faced increased challenges compounded by diminished service availability and individual ability to influence their situations including access to basic provisions, being able to sustain the costs of daily living, residing in inappropriate accommodation and struggling with the absence of both practical and emotional support. While some young people felt supported, others felt neglected and forgotten Roberts, Rees et al. (2021).

Cross‐cultural studies and studies conducted among minorities have emphasized the differing routes into adulthood among young adults from various socioeconomic statuses and ethnicities (e.g. Helve & Bynner, 2007; Syed & Mitchell, 2013) and have suggested that the transition to adulthood among these individuals might be more challenging than that of their peers (Arnett, 2016; Ibrahim & Howe, 2011; Phinney, 2006). For example, Côté (2002) found that the greatest transitional difficulties befell those with the fewest economic, intellectual and psychological resources. The current study's findings add to the existing literature and present COVID‐19 as an additional difficulty, over and above the challenges already faced by these vulnerable young adults. Moreover, the findings reveal that for these individuals, the pandemic has not been just another “challenge” to contend with but a profound component that has affected most of the areas comprising their daily lives and their future plans. It is eminently clear that the pandemic held back at‐risk young adult Arabs who had previously begun to provide for themselves and further worsened the situations of those who had been struggling just to survive. Some of the young adults with a history of maltreatment emphasized their complex relationships with their families and the influence it had on them mentally. Feeling of safety and belonging were also found among other groups of vulnerable young adults during COVID‐19 who felt that being confined to their ‘unsafe’ home amplified their anxieties (Roberts, Mannay, et al., 2021).

In contrast to Western societies that tend to place a greater emphasis on the individual, the self in a collectivist society is not an autonomous being but, instead, connected to an in‐group (Dwairy et al., 2006). As such, individuals' identities in collectivist cultures cannot easily be separated from their family's identity. Indeed, the current study's findings support this idea and indicate that this perspective places at‐risk young adult Arabs in a complex reality, generally, and specifically during the era of COVID‐19. Namely, we found that many of the participants were committed to their families and felt obligated to support them in various ways, including by supporting parents and siblings financially during this global health crisis. The central place held by such cultural values in a patriarchal, family‐based and honour‐bound society was also identified in earlier studies among at‐risk young adult Arabs in Jordan (Ibrahim & Howe, 2011) and in Israel (Sulimani‐Aidan, 2020a) wherein young adults described the influence of cultural and social expectations, as well as their national minority status, on their future plans and self‐perceptions.

The collectivist nature of Arab society may be perceived as an asset for young adults in that it can give them a sense of belonging and a source of support, emotionally and materially, throughout life and in particular during transitional periods (Dwairy et al., 2006; Sulimani‐Aidan, 2020b). However, it may also serve as an additional risk factor especially among families from low socioeconomic status (Shoshana, 2020) and young women who suffer from further cultural restrictions (Sulimani‐Aidan, 2020a). The findings of the current study align with these earlier findings and demonstrate the additional burden placed on these young adults as a result of their ‘cultural devotion’. Although the majority of the participants in the current study lived with their parents, it was evident that they were in need of additional emotional and material support during this period. Nevertheless, they postponed and neglected their own needs in order to meet the heightened needs of their families (i.e. as a result of the pandemic). Accordingly, the main features that ordinarily typify the period of emerging adulthood, such as self‐exploration, self‐focus and making plans for the future (Arnett, 2004) were found to be not only limited, as found in previous studies conducted among young adults of ethnic minorities (Arnett, 2016; Ibrahim & Howe, 2011; Phinney, 2006; Sulimani‐Aidan, 2020a) but actually put off indefinitely. The need to adhere to collectivist values (in line with familial and cultural expectations) and at the same time seek self‐exploration and ponder future possibilities (in line with Western expectations of emerging adults) seems to lead to constant tension. Although prior to the pandemic many of the young adults seemed to find ways to manage this tension, the arrival of the pandemic threw their entire futures into question. It is worth mentioning that the pandemic's impact on the lives of these young adults was integrative‐circular, meaning that each area that was influenced by the pandemic affected another area and so on. The intersection between these young adults' poor personal and familial assets, structural vulnerability and sociocultural characteristics seemed to lead to further strain, vulnerability and risk during the pandemic. This reality seems to have amplified the instability and uncertainty that characterizes the period of emerging adulthood (Arnett, 2000).

### 5.2 Limitations and future directions

This study provides initial insights into the unique experiences of at‐risk Israeli‐Arab young adults during the transition to adulthood in the wake of COVID‐19. However, a few limitations should be acknowledged. First, as an exploratory study, only a small convenience sample was used. The study's sample does not represent all Israeli‐Arab young adults. Therefore, one should be cautious when generalizing from these findings. Also, further exploration is needed in order to shed more light on the differential experiences resulting from various characteristics within the population, as it is not a monolithic one. For example, we did not distinguish between Muslim‐Arabs, Christian‐Arabs, or Druze, nor did we look at gender differences. Also, further studies should include the perspectives of those who have cared for/worked with/supported these young adults, such as caseworkers and mentors. Their inclusion could provide a more comprehensive understanding regarding the needs of this cohort during this period. In addition, further comparative studies among groups of at‐risk young people versus their peers in the general populations could shed more light on the themes that emerged including relationship with family members and social network. Finally, longitudinal studies via the use of both qualitative and quantitative methods are needed to assess and understand the long‐term consequences of the pandemic for these young adults' future paths.

### 5.3 Implications for policy and practice

The study's findings illustrate the multiple challenges and barriers faced by at‐risk young adult Arabs as emerging adults during a global pandemic and call for a deeper acknowledgment of their unique experiences as a national minority group.

The findings also indicate the need for additional resources to support them during their transition to adulthood. One of the main aspects that should be considered in terms of policy and practice is their occupational and financial status. Reducing poverty among the Arab minority in Israel, both generally and in the wake of the pandemic, is crucial in order for these young adults to make their way in life. Therefore, in order to lessen the consequences of financial strain and unemployment, additional public support is essential. Increasing opportunities to participate in the labor market may enhance these vulnerable young people's chances to experience social inclusion, with tangible social and psychological gains (Gilligan, 2019).

Although the majority of the study participants did not specifically indicate that the pandemic had influenced their mental health, their descriptions regarding their constant financial strain, conflicts within the family, worries about their future and loneliness elicit concerns regarding their psychological distress during this time. Research has already shown that, as part of a minority group, they are exposed to more stressful life events and higher social stress than are their Jewish counterparts (Daoud et al., 2012). The worldwide lockdown and the restrictions imposed on social gatherings in the wake of the pandemic have increased and strengthened feelings of loneliness, particularly among young adults, causing damage to their mental health (Serafini et al., 2020; WHO, 2020). Those feeling of loneliness and isolation were also documented in the few earlier studies conducted among at‐risk young adult Arabs ahead of the pandemic outbreak (Sulimani‐Aidan, 2020a, 2020b). Indeed, a sense of isolation, loneliness and lack of guidance and emotional support were also dominant in the current study participants' descriptions. Clearly, there is a need for psychological and social awareness given this pandemic. Also, it is imperative that services commit to increased resources to support this groups needs and to ensure the provision of appropriate interventions and provide responsive mental health services, so that they would feel protected and supported.

Finally, the study's findings reveal that many areas are interwoven, and the needs of these at‐risk young adults are broad. Therefore, it is important to design holistic services that address their needs while taking into account the collectivist nature of Arab society.
